# Effects of high-intensity interval training on biomarkers and inflammatory factors in patients with heart failure: a meta-analysis of randomized controlled trials

**DOI:** 10.3389/fcvm.2025.1641635

**Published:** 2025-10-17

**Authors:** Wanting Zhang, Jooyeon Hwang, Beibei Lu, Xin Xu

**Affiliations:** ^1^ School of Exercise and Health, Shanghai University of Sport, Shanghai, China; ^2^ Department of Environmental & Occupational Health Sciences, School of Public Health, University of Texas Health Science Center, Houston, TX, United States; ^3^ Southwest Center for Occupational and Environmental Health, University of Texas Health Science Center, Houston, TX, United States; ^4^ Research Institute for Doping Control, Shanghai University of Sport, Shanghai, China

**Keywords:** high-Intensity interval training, heart failure, cardiac rehabilitation, inflammatory biomarkers, meta-analysis

## Abstract

**Background:**

There is still controversy about the effect of high-intensity interval training (HIIT) on the levels of cardiac and inflammatory biomarkers in patients with heart failure compared with moderate-intensity continuous training (MCT) and conventional healthcare activities. This article is to systematically investigate the effects of HIIT on the levels of cardiac and inflammatory biomarkers in patients with heart failure compared with MCT and conventional healthcare activities.

**Methods:**

A computerized search of databases was conducted with the search year from the establishment of the database, and the screened results were subjected to quality assessment and data extraction, and the data was meta-analyzed using RevMan 5.4.1 software.

**Results:**

11 randomized controlled trials (RCTs) were finally included (7 RCTs about BNP and NT-proBNP, 2 RCTs about inflammatory factors, and 2 RCTs involve both), containing a total of 904 study subjects, 457 in the observation group, and 447 in the control group. Meta-analysis showed that HIIT significantly reduced BNP and NT-proBNP levels in patients with heart failure compared with the control group (SMD = −1.33, 95% CI: −2.55∼−0.11, *P* = 0.03), while it had no significant effect on the level of CRP (SMD = −0.08, 95% CI: −0.33∼0.18, *P* = 0.56), TNF-α (MD = −0.11, 95% CI: −0.29∼0.08, *P* = 0.26), and IL-6 (MD = 0.3, 95% CI: −1.95∼2.55, *P* = 0.8).

**Conclusions:**

Compared with MCT and conventional healthcare activities, the implementation of HIIT in patients with heart failure can effectively reduce the levels of heart failure biomarkers and improve their health. However, the difference between HIIT and the control group was not significant in the regulation of inflammatory factors.

**Systematic Review Registration:**

https://www.crd.york.ac.uk/PROSPERO/#searchadvanced, PROSPERO CRD42024513099.

## 1 Introduction

Heart Failure often occurs in the end stages of many types of cardiac diseases and has become a sanitation problem that seriously threatens human's health. According to the latest epidemiological studies in China, the current prevalence of heart failure is 1.1%, and the hospitalization cost reaches $ 4,406.8 US dollars per capita (1). Patients with heart failure often suffer from a huge medical and socio-economic burden. Therefore, reasonable and effective rehabilitation is very necessary for heart failure patients.

Cardiac Rehabilitation (CR) is an integrated disciplinary program that focuses on exercise training, supplemented by psychosocial assessment, medication adherence education, stress reduction counseling, and risk factor modification (2). It has been classified by the European Society of Cardiology (ESC) as a level-I recommended measure for the prevention and treatment of the cardiovascular disease (3). For a long time, numerous studies have demonstrated the safety and benefits of exercise for patients with heart failure (4–6). A large-scale exercise intervention study called HF-ACTION showed that exercise training reduced the risk of all-cause mortality or hospitalization by 11% and cardiovascular-related mortality and morbidity by 15% (7).

The target intensity of High-Intensity Interval Training (HIIT) is between 80% and 100% of maximum heart rate or aerobic capacity (8). In comparison, Moderate-Intensity Continuous Training (MCT) targets 55%–70% of heart rate (9). HIIT is characterized by brief intermittent bursts of vigorous activity (10). Based on these differences, the divergent effects of HIIT and MCT on cardiac rehabilitation in heart failure patients have drawn increasing attention. Therefore, this study uses the classic cardiac biomarkers Brain natriuretic peptide (BNP) and N-terminal pro-B-type natriuretic peptide (NT-proBNP) as key indicators for assessing heart failure, aiming to investigate whether HIIT is more effective for cardiac rehabilitation.

Additionally, inflammatory factors play a crucial role in the pathogenesis of heart failure (11). Studies have found that inflammatory factors interact with BNP/NT-proBNP during the pathological process (12), and monitoring both markers together allows for a more precise evaluation of HF severity than using either indicator alone (13). Although a growing number of studies have demonstrated the effects of HIIT on maximal oxygen uptake (VO₂ max), stroke volume, vascular function, and mitochondrial adaptation (14–16), the specific ways in which HIIT regulates inflammatory cytokine levels in the body remain unclear. Based on this, this study employs a meta-analytic approach to investigate whether the cardiac rehabilitation mechanisms of HIIT are associated with changes in inflammatory factors levels, thereby providing more evidence for exercise rehabilitation strategies in heart failure patients.

## 2 Methods

This study followed the prescribed process of the Preferred Reporting Items for Systematic Reviews and Meta-Analysis (PRISMA) (Supplementary Table S1) (17) and completed protocol registration on the PROSPERO platform (registration number: CRD42024513099).

### 2.1 Search strategy

The literature search was performed on PubMed, Web of Science, The Cochrane Library, Embase, EBSCO, CNKI, Wanfang, and VIP databases. The search year is from the year of construction to May 1st, 2025. Based on the PICOS principle and Boolean logic to conduct the search strategy. Search terms: (“Heart Failure” OR “Cardiac Failure” OR “Myocardial Failure” OR “Cardiac Insufficiency”) AND (“High-Intensity Interval Training” OR “High-Intensity Intermittent Exercise” OR “HIIT”) AND (“Inflammation”) AND (“Randomized” OR “Placebo”).

Taking PubMed as an example, the specific search strategy is shown as follow:

#1 “Heart Failure” [MeSH]

#2 “Heart Failure” OR “Cardiac Failure” OR “Myocardial Failure” OR “Cardiac Insufficiency” OR “Heart Decompensation” OR “Decompensation, Heart” OR “Congestive Heart Failure” OR “Heart Failure, Congestive”[Title/Abstract]

#3 #1 OR #2

#4 “High-Intensity Interval Training” [MeSH]

#5 “High-Intensity Interval Training” OR “High Intensity Interval Training” OR “High-Intensity Interval Trainings” OR “Interval Training, High-Intensity” OR “Interval Trainings, High-Intensity” OR “Training, High-Intensity Interval” OR “Trainings, High-Intensity Interval” OR “High-Intensity Intermittent Exercise” OR “Exercise, High-Intensity Intermittent” OR “Exercises, High-Intensity Intermittent” OR “High-Intensity Intermittent Exercises” OR “Sprint Interval Training” OR “Sprint Interval Trainings” OR “HIIT” OR “HIIE” [Title/Abstract]

#6 #4 OR #5

#7 “Inflammation” [MeSH]

#8 “Inflammation” OR “Inflammations” OR “Innate Inflammatory Response” OR “Inflammatory Response, Innate” OR “Innate Inflammatory Responses” [Title/Abstract]

#9 #7 OR #8

#10 #3 AND #6 AND #9 [Filter: Randomized Controlled Trial]

### 2.2 Selection criteria

#### 2.2.1 Inclusion criteria

1. Study type: published randomized controlled trial (RCT);
2. Study subjects: patients clinically diagnosed with heart failure, with no restriction on gender, age, race, and ethnicity;
3. Interventions: High-intensity interval training (HIIT) in the test group and moderate-intensity continuous exercise or routine healthcare activities in the control group;
4. Outcome indicators: Brain natriuretic peptide (BNP) and N-terminal pro-B-type natriuretic peptide (NT-proBNP), C-reactive protein (CRP), tumor necrosis factor-alpha (TNF-α), interleukin-6 (IL-6).

#### 2.2.2 Exclusion criteria

1. Non-RCT literature such as reviews, meta-analyses, systematic evaluations, conference proceedings, clinical cases, etc;
2. Repeatedly published;
3. Animal experiment literature;
4. Heart failure combined with other injuries or complications;
5. Combination of intervention with other therapies;
6. Incomplete information, incomplete experimental procedures, or insufficient experimental data;
7. Inaccessible full text;
8. Literature not in Chinese or English (Table 1).

### 2.3 Data extraction

The literature search was first carried out in the database using the identified search strategy, and then the literature screening process was carried out using EndNote21 software. Some irrelevant subject matter literature was excluded by reading the title and abstract of the literature, and then non-compliant literature was gradually excluded according to the inclusion and exclusion criteria. This process was done independently by the two researchers (Zhang W.T. and Xu X.), and when differences of opinions occurred, disagreements were resolved by a third reviewer.

**Table 1:** Inclusion and exclusion criteria.

Characteristic	Inclusion	Exclusion
Participants	Patients clinically diagnosed with heart failure, with no restriction on gender, age, race, and ethnicity	Heart failure combined with other injuries or complications
Intervention	High-intensity interval training (HIIT)	Combination of intervention with other therapies
Comparison	Moderate-intensity continuous exercise (MCT) or conventional healthcare activities	Other forms of comparison
Outcomes	Brain Natriuretic Peptide (BNP) and N-Terminal pro-B-type Natriuretic Peptide (NT-proBNP); C-Reactive Protein (CRP); Tumor Necrosis Factor-Alpha (TNF-alpha); Interleukin-6 (IL-6)	1) Incomplete information, incomplete experimental procedures, or insufficient experimental data; 2) Animal experiment literature
Study Design	Published randomized controlled trial (RCT)	Non-RCT literature such as reviews, meta-analyses, systematic evaluations, conference proceedings, clinical cases, etc
Others		1) Repeatedly published; 2) Inaccessible full text; 3) Literature not in Chinese or English

Data extraction included the first author, date of publication, age of patients, sample size, New York Heart Association (NYHA) Classification of patients, gender ratio of patients, form of exercise intervention, intensity of exercise, frequency of exercise, duration of exercise, period of intervention, and outcome indicators.

### 2.4 Quality assessment

The risk of bias evaluation of the included literature was performed using the Cochrane Collaboration's tool for assessing risk of bias (18).

Evaluation entries included: randomized sequence generation, allocation concealment, blinding of outcome assessors, incomplete outcome, selective outcome reporting, and other risks of bias including, but not limited to, the sample size being too small (<10), and unsupervised exercise regimens. The item “blinding of participants and personnel” was not evaluated because double-blinding of implementers and participants is impractical to achieve in randomized controlled trials of exercise interventions.

### 2.5 Statistical analysis

RevMan 5.4.1 software was used to analyze the data statistically. The weighted mean difference (WMD) or standardized mean difference (SMD) was used as the effect analysis statistic for the measurement data. WMD was used as the effect indicator when the evaluation methods and units of the outcome indicators were the same while SMD was used as the effect indicator when the evaluation methods and units of the outcome indicators were different, and the respective effect sizes was expressed as 95% confidence interval (CI). When the mean or standard deviation (SD) were not shown in the original literature, the following methods were used for data conversion: 1) Estimating the mean and SD from the median and maximum and minimum values; 2) Estimating the mean and SD from the median and quartiles; 3) Intercepting the data from statistical graphs; 4) Estimating the SD by the standard error (SE) (19).

The heterogeneity analysis was performed using *I*^2^. When *I*^2^ < 50% and *P* > 0.1, the heterogeneity among studies was considered small, and fixed-effects model was used to combine effect sizes. When *I*^2^ > 50% or *P* < 0.1, the heterogeneity among studies was considered large, and random-effects model was used to combine effect sizes. When there was significant clinical heterogeneity, subgroup analysis or sensitivity analysis was used for treatment. If data could not be combined between studies, descriptive analysis was used. Significant statistical differences between groups were expressed as *P* < 0.05.

## 3 Results

### 3.1 Search results

**Figure 1:**
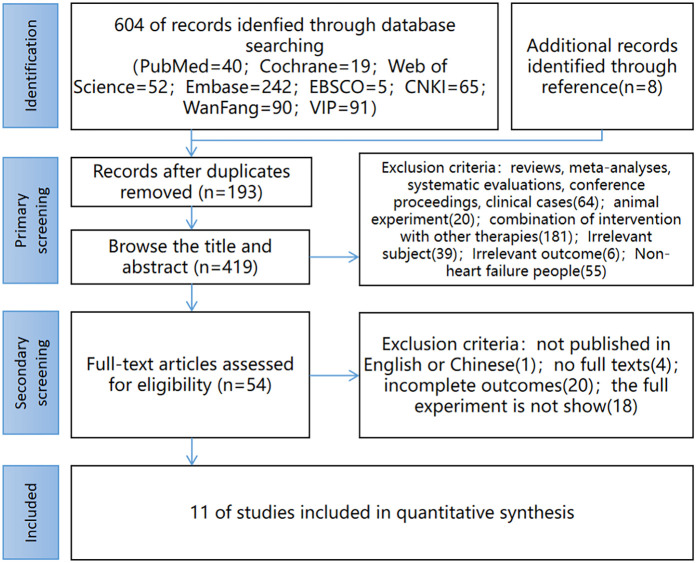
Study selection flow diagram. (CNKI China National Knowledge Infrastructure; Wanfang Wanfang Database; VIP China Science and Technology Journal Database).

604 literature were retrieved from eight databases, and 8 literature were obtained from other sources, totaling 612. After eliminating duplicates, 419 literature remain, 54 literature remain after initial screening through the title and abstract, and 11 literature finally remain after re-screening by reading the full text. The literature screening process is shown in Figure 1.

### 3.2 Study characteristics

11 RCTs were included, containing a total of 904 study subjects, 457 in the observation group, and 447 in the control group. The basic characteristics of the included literature are shown in Table 2.

**Table 2:** Characteristic of studies included.

First author		Participants				Interventions					
	Country	Age (y, mean ± SD)	Sample size (*n*)	NYHA (II/III)	Sex (M/F)	Form	Intensity	Frequency (time/week)	Duration (min/time)	Period	Outcomes
Papathanasiou 2022 (20)	Bulgaria	C 63.82 ± 6.71	60	46/14	35/25	MCT	70% HR max	4	40	12 Week	①③
		T 63.65 ± 6.71	60	48/12	35/25	HIIT	90% HR max				
Gao 2022 (21)	China	C 64.38 ± 1.26	48	22/26	25/23	MCT	50%–70% HR max	3	60	12 Week	②
		T 64.43 ± 1.28	48	23/25	27/21	HIIT	80%–90% HR max		40		
Mueller 2021 (14)	Germany Belgium Norway	C 70 ± 8	58	44/14	23/35	MCT	35%–50% HRR	5	40	12 Month	②
		T 70 ± 7	58	44/14	17/41	HIIT	Warm-up: 35%-50% HRR Main Part: 80%-90% HRR	3	38		
Mao 2020 (22)	China	C 61 ± 1.5	83	69/14	68/15	MCT	70%-75% VO_2_ peak	3	30	12 Week	②
		T 64 ± 2.5	81	63/18	66/15	HIIT	Main Part: 90%–95% VO_2_ peak Interval: 60%–70% VO_2_ peak		38		
Chou 2018 (23)	China	C 59.7 ± 5.3	15	Unknown	11/4	NE	–	–	–	12 Week	②
		T 60.9 ± 0.5	15		11/4	HIIT	40%–80% VO_2_ peak	3	30		
Ellingsen 2017 (15)	Europe (country unknown)	C 60 ± 1.75	65	41/24	53/12	MCT	60%–70% HR max	3	47	12 Week	②
		T 65 ± 2.5	77	55/22	63/14	HIIT	90%–95% HR max		38		
Aksoy 2015 (24)	Turkey	C 59.6 ± 6.9	15	Unknown	13/2	MIC	50%–75% VO_2_ peak	3	35	10 Week	①②
		T 63.7 ± 8.8	15		13/2	HIIT					
Fu 2013 (25)	China	C 67.5 ± 1.8	15	Unknown	9/5	MCT	Warm-up: 30% VO_2_ peak (3 min) Main Part: 60% VO_2_ peak (30 min) Cold-down: 30% VO_2_ peak (3 min)	3	30	12 Week	②④
		T 66.3 ± 1.2	15		8/5	HIIT	Warm-up: 30% VO_2_ peak (3 min) Main Part: 80% VO_2_ peak (3min)+ Interval 40% VO_2_ peak (3 min), 3 times Cold-down: 30% VO_2_ peak				
Byrkjeland 2011 (26)	Norway	C 71.5 ± 7.8	40	24/16	33/7	NE	–	–	–	4 Month	①③④
		T 68.8 ± 7.9	40	21/19	30/10	HIIT	Main Part: Brog 15–18 Interval: Brog 11–13	2	50		
Nilsson 2010 (27)	Norway	C 71.5 ± 7.9	39	24/15	31/8	NE	–	–	–	4 Month	②
		T 68.8 ± 7.9	39	21/18	30/9	HIIT	Brog 15–18	2	50		
WislOff 2007 (16)	Norway	C 74.4 ± 12	9	Unknown	7/2	MCT	70%–75% HR peak	3	47	12 Week	②
		T2 76.5 ± 9	9		7/2	HIIT	Warm-up: 60%–70% HR peak Main: 90%–95% HR peak + Interval 50%–70% HR peak Cold-down: 50%–70% HR peak	3	38		

Brog, Borg scale; C, control group; F, female; HIIT, high-intensity interval training; HR max, maximum heart rate; HRR, heart rate reserve; HR peak, peak heart rate; M, male; MCT, moderate-intensity continuous training; NE, no exercise; NYHA, classification of New York Heart Association heart function; T, training group; VO2 peak, peak oxygen uptake; ①, BNP or NT-pro BNP; ②, CRP; ③, TNF-α; ④, IL-6.

**Figure 2:**
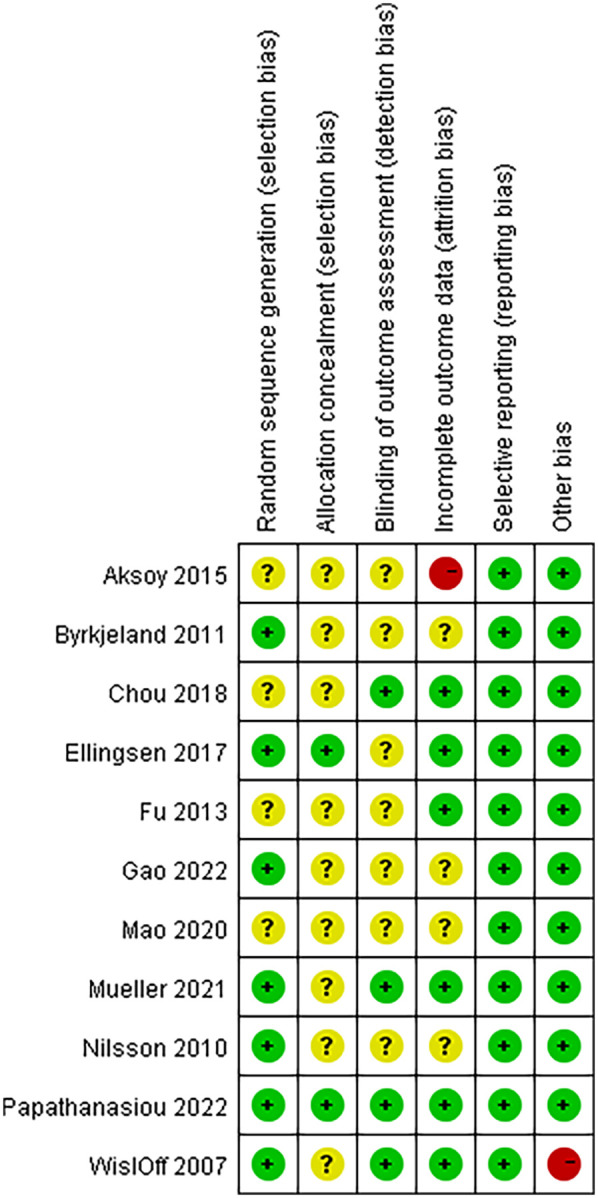
Details of the study quality assessment according to Cochrane collaboration risk-of-bias tool.

The Cochrane Collaboration's tool for assessing risk of bias was used to evaluate the quality of the above literature. The evaluation results are shown in Figure 2.

### 3.3 Meta-analysis results

#### 3.3.1 Biomarkers of heart failure

**Figure 3:**
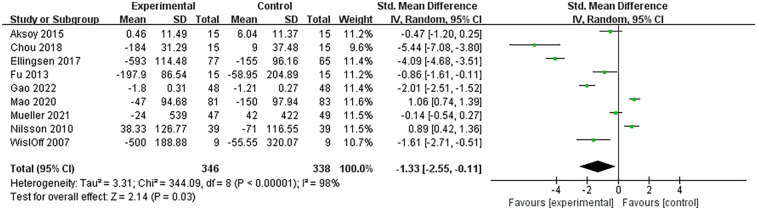
Forest plot of the effects of HIIT on BNP and NT-proBNP in patients with heart failure.

The biomarkers of heart failure involved in this study included BNP and NT-proBNP. A total of 9 RCTs analyzed the levels of these two biomarkers and included 684 patients with heart failure. The heterogeneity among studies was large (*I*^2^ = 98%, *P* < 0.00001), so meta-analysis was performed using a random-effects model. The results showed that HIIT significantly reduced the levels of biomarkers in patients with heart failure compared with the control group (SMD = −1.33, 95% CI: −2.55 to −0.11, *P* = 0.03). Specifically, the SMD value of BNP/NT-proBNP was −1.33, indicating that biomarkers in the HIIT group were lower than those in the control group. According to Cohen's standards for small, medium and large effect sizes, the |SMD| = 1.33 > 0.8 is considered a large effect size, suggesting that the reduction in HIIT has a significant effect. Combined with a *P* value of 0.03 (indicating a statistical difference), it suggests that compared to the MCT and conventional healthcare activities, BNP and NT-proBNP were more significantly cleared in the HIIT group. The 95% CI was −2.55 to −0.11 (not crosses 0), indicating that this difference was not caused by random error, and the result was statistically significant (Figure 3).

#### 3.3.2 Inflammatory factors

**Figure 4:**
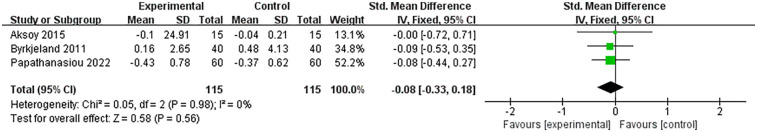
Forest plot of the effects of HIIT on CRP in patients with heart failure.

**Figure 5:**
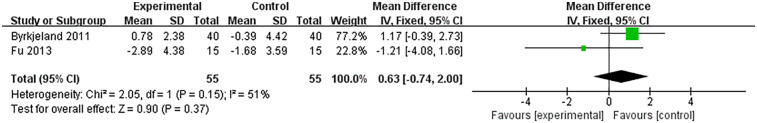
Forest plot of the effects of HIIT on TNF-α in patients with heart failure.

Inflammatory factors involved in this study included CRP, TNF-α, and IL-6. 3 RCTs analyzed the levels of CRP and included 230 patients with heart failure. The heterogeneity between studies was small (*I*^2^ = 0%, *P* = 0.98), so a fixed-effects model was used for meta-analysis. The meta-analysis results are: SMD = −0.08, 95% CI: −0.33 to 0.18, *P* = 0.56 (Figure 4). And 2 RCTs analyzed the levels of TNF-α and included 178 patients with heart failure. The heterogeneity between studies was small (*I*^2^ = 36%, *P* = 0.21), so a fixed-effect model was used for meta-analysis. The meta-analysis results are: MD = −0.11, 95% CI: −0.29 to 0.08, *P* = 0.26 (Figure 5). The SMD of CRP was −0.08, and the MD of TNF-α was −0.11, both indicated that the outcome indicators of the HIIT group were lower than those of the control group. This means HIIT was more effective than the MCT or regular healthcare group in clearing CRP and TNF-α in patients with HF. However, the *P* values for both indicators were greater than 0.05, and the 95% CI for both crossed 0 (from negative to positive), indicating that this slight downward trend did not reach statistical significance. This might be related to the small number of studies included in the meta-analysis.

**Figure 6:**
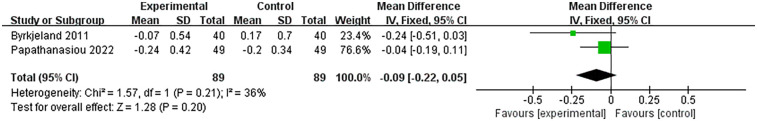
Forest plot of the effects of HIIT on IL-6 in patients with heart failure.

2 RCTs analyzed the levels of IL-6 and included 120 patients with heart failure. The heterogeneity among studies was large (*I*^2^ = 51%, *P* = 0.15), so meta-analysis was conducted using a random-effects model. The meta-analysis results are: MD = 0.3, 95% CI: −1.95 to 2.55, *P* = 0.8 (Figure 6). The MD value of IL-6 was 0.3, and the 95% CI range was −1.95 to 2.55, with a *P* value greater than 0.05, indicating that IL-6 slightly increased in the HIIT group, but this trend did not reach a significant difference. This might be related to the dual effects of IL-6.

### 3.4 Sensitivity analysis and publication bias

Sensitivity analysis was performed on the meta results of biomarkers to find the source of heterogeneity. After excluding individual studies one by one, no single study was found to significantly affect the heterogeneity, so the overall stability of the study results was good.

The publication bias test was performed using the Begg test which shows that the *P* value of each outcome indicator was greater than 0.05 (heart failure markers: *P* = 0.061, CRP: *P* = 0.602, TNF-α: *P* = 0.317, and IL-6: *P* = 0.317), and therefore the possibility of publication bias was low. (A funnel plot of publication bias for biomarkers is presented in Supplementary Figure S1).

## 4 Discussion

The main finding of this study was that HIIT can significantly reduce the levels of natriuretic peptide biomarkers (BNP and NT-proBNP) in patients with heart failure (SMD = −1.33, 95% CI: −2.55 to −0.11, *P* = 0.03), proving that HIIT may have better efficacy in the rehabilitation of heart failure patients.

The World Health Organization (WHO) defined biomarkers as any substance, structure, or process that can be measured *in vivo* or its products and that can have an effect on, or be predictive of, outcome and disease incidence (28). Thus, biomarkers have been widely used in the diagnosis, clinical assessment, and prognostic evaluation of heart failure (29). Among them, the persistent elevation of brain natriuretic peptide (BNP) and N-terminal B-type natriuretic peptide proteins (NT-proBNP) are regarded as the important indicators for assessing heart failure. They have been used in clinical practice for over 20 years and are recommended as level I recommendation in multiple clinical practice guidelines (3, 30). Both BNP and NT-proBNP belong to the family of natriuretic peptides. They are mainly synthesized and secreted by ventricular myocytes, which are increased in the presence of ventricular overload or ventricular dilatation, and can be used as a specific sensitivity indicator to reflect ventricular disorders (31). Due to the highly overlap in their biological effects, and considering the limited number of included articles, in order to avoid a decrease in statistical efficiency caused by the detailed classification of indicators, this study combined BNP and NT-proBNP for research.

This meta-analysis summarized 9 RCTs on heart failure markers. Among them, Gao et al. found that the level of NT-proBNP was reduced in the body of patients with heart failure after both HIIT and MCT, but the reduction of NT-proBNP level after HIIT was slightly more than after MCT, demonstrating superior efficacy of HIIT (21). But Mao et al. concluded the opposite that MCT would be slightly more effective than HIIT in reducing the level of NT-proBNP (22).

A similar controversy was found in the controlled trials of HIIT vs. conventional health care. Chou et al. found that BNP level was reduced in patients with heart failure who underwent HIIT, whereas BNP level was elevated in patients who only underwent conventional health care activities, demonstrating the benefits of HIIT on the recovery of patients with heart failure (23). In contrast, Nilsson et al. found that NT-proBNP level was elevated after HIIT, but decreased in patients who performed only conventional healthcare activities, and HIIT may have induced adverse effects (27).

This meta-analysis revealed that, although controversial, the results of most studies tended to favor HIIT as more beneficial for cardiac rehabilitation. Eillngsen et al. and Wisløff et al. found significant reductions in NT-proBNP level after HIIT relative to MCT (15, 16). Fu et al. found significant reductions in BNP level after HIIT relative to MCT (25). While Mueller et al. found that the level of NT-proBNP was reduced in patients after HIIT, while it was elevated after MCT (14). And Aksoy et al. found that NT-proBNP level was elevated after both HIIT and MCT, but the elevation was relatively lesser after HIIT (24).

To determine whether the high heterogeneity of the results is related to the intensity and duration of the exercise intervention, we conducted a sensitivity analysis. Among the included articles, the intervention period of most studies was 10–16 weeks, and only one RCT had an intervention period of 48 weeks (Mueller et al.). The training intensity of each study varied, for example, Aksoy et al. used 50%–75% HR as the standard for HIIT training, but Mao et al. used 90%–95% HR as the training standard. The duration of each single intervention in all studies was similar, ranging from 30 to 50 min. After eliminating each article one by one, the heterogeneity did not change significantly, proving that there is no single study influencing the heterogeneity of the results.

Another finding of this study concerns the changes in inflammatory factors, including CRP, TNF-α, IL-6:

Inflammatory responses occur throughout heart failure (11). Inflammatory mediators play an important role in the pathogenesis of heart failure, and that inflammation is perhaps one of the drivers of ventricular remodeling that influences the progression of heart failure (32). The ability of multiple inflammatory markers to predict progression is independent of traditional disease predictors. First of all, CRP levels are one of the markers of tissue damage. Radenovic et al. showed that elevated CRP levels were positively associated with poor outcomes in heart failure patients (33). Secondly, TNF-α can promote the progression of heart failure by mediating oxidative stress causing cardiomyocyte hypertrophy, apoptosis, and fibrosis. Dunlay et al. found that higher levels of TNF-α in patients with heart failure were associated with survival rates were negatively correlated (34). Thirdly, IL-6 can exert negative inotropic effects through the gp130/STAT3 pathway, thereby promoting cardiomyocyte hypertrophy and fibrosis. A study by Gwechenberger et al. found that IL-6 was consistently up-regulated in a heart failure model of cardiac injury (35). Therefore, reducing levels of CRP, TNF-α, and IL-6 in heart failure patients may help to reduce heart failure symptoms and improve the overall health of heart failure patients. In view of these properties, CRP, TNF-α and IL-6 were selected for the study.

A total of 4 RCTs which were included in this meta-analysis involved CRP, TNF-α and IL-6.

Papathanasiou et al. and Aksoy et al. found that CRP level was reduced in patients with heart failure after both HIIT and MCT, and HIIT having a slightly better effect than MCT (20, 24). Differently, Byrkjeland et al. found that CRP level was elevated after either HIIT or conventional healthcare activities, but the magnitude of the HIIT elevation was relatively lesser (26).

In addition, TNF-α level was found to be reduced after both HIIT and MCT in Papathanasiou et al. (23). And Byrkjeland et al's findings further add to the conclusion that TNF-α level was elevated in patients who performed only conventional healthcare activities (26).

Fu et al. found that IL-6 was reduced in patients with heart failure after both HIIT and MCT, and HIIT was slightly more effective than MCT (25). But Byrkjeland et al. found the opposite result, arguing that IL-6 level was elevated after HIIT, and that instead, conventional healthcare activities promoted a reduction in IL-6 level (26).

The difference in the direction of this change in IL-6 may be related to its dual effects of both pro-inflammatory and anti-inflammatory. Previous studies have confirmed that IL-6 can promote the generation of inflammatory factors by activating the JAK/STAT pathway and TGF-β-related pathways, and can also inhibit excessive inflammatory responses by regulating the mblL-6R receptor, Th17/Treg cells, dendritic cells, IL-10, etc. (36). In addition, differences in exercise intensity, exercise mode, and the interval of post-exercise detection may also lead to high heterogeneity in IL-6 results (37).

Due to the limitation of space, only a few common inflammatory factors were selected for discussion in this study, but in recent years, with the development of basic experimental methods and techniques, more and more inflammatory factors have been found to provide diagnostic and predictive information of heart failure. For example, interleukin-1 (IL-1), interleukin-10 (IL-10), interleukin-18 (IL-18), fibrinogen, inducible nitric oxide synthase (iNOS), myeloperoxidase (MPO), etc (38). The effect of exercise on the levels of other inflammatory factors in heart failure patients can be further explored in the future.

For a long time, MCT is the most commonly chosen exercise program for patients with heart failure, but this exercise form is relatively single-form, exercise time-consuming, and the short-term benefits are not obvious, which makes it difficult for a large number of patients to adhere to exercise. In contrast, HIIT takes a much shorter time with the same total energy consumption and has various forms and contents, which can improve the cardiopulmonary function and exercise endurance of patients in the short term, so it is easier for patients to adhere to and benefit from HIIT (39, 40).

In order to explore in depth the similarities and differences between HIIT and MCT, as well as to better explain this hypothesis, deeply investigate the pathogenesis of heart failure, search for specific biomarkers, explore new therapeutic targets, and improve existing treatment options, in addition to meta-analysis, the effect of HIIT on heart failure and cardiac rehabilitation can be further analyzed in conjunction with multi-omics techniques.

Over the past two decades, the emergence and development of multi-omics techniques have greatly facilitated the research process in cardiac pathophysiology (41, 42). In the last few years, multi-omics techniques have also gradually been widely used in the field of sports (43, 44). Among them, metabolomics is one of the latest omics technologies that can effectively integrate genomics, transcriptomics and proteomics, provides quantitative information on exercise-related metabolic profiles to identify key biomarkers associated with exercise performance and potential exercise-related diseases (45, 46).

Because cardiovascular disease is a complex pathological process with multifactorial involvement, research on biomarkers should also be multilevel. Currently, our group has published articles revealing the multi-omics' characteristics of high-intensity interval training (47). And in the future, we hope to publish further articles on biomarkers identifying inflammatory factors in heart failure. And wherever possible, these studies were combined to explore in depth the pathways and mechanisms of exercise interventions in the heart failure pathway. To address new strategies for the development of CR and exercise intervention, in the future, more and more detailed RCT studies should be linked to multi-omics technologies and basic experiments to improve the prediction of heart failure risk, and establish simpler, more accurate, efficient, and safer diagnostic methods, and provide patients with more targeted and effective therapeutic and prognostic options.

The limitations of this meta-analysis are summarized as follows: 1) Some of the included studies did not explicitly state whether they implemented random allocation, allocation concealment scheme, or blinding, which may pose a risk of bias in implementation, measurement, and assessment; 2) The included studies were not fully harmonized in terms of exercise intensity, intervention period, and exercise strategy, which resulted in the heterogeneity of multiple outcome indicators; 3) The number of RCTs included in some of the outcome indicators was relatively small.

## 5 Conclusions

Compared with moderate-intensity continuous exercise and conventional healthcare activities, the implementation of high-intensity interval training can effectively reduce the levels of biomarkers in patients with heart failure and improve their health. However, the difference between HIIT and the control group was not significant in the regulation of inflammatory factors, such as CRP, TNF-α and IL-6. Due to the limitations of the number and quality of the included studies, the above conclusions are subject to further validation.

## Data Availability

The original contributions presented in the study are included in the article/Supplementary Material, further inquiries can be directed to the corresponding author.

## Supplementary material

Supplementary Figure S1 Funnel plot of the publication bias of the included studies about biomarkers.
