# Supplementary figures and images for: Effects of high-intensity interval training on biomarkers and inflammatory factors in patients with heart failure: a meta-analysis of randomized controlled trials
Source: Front Cardiovasc Med. 2025 Oct 17;12:1641635. doi: 10.3389/fcvm.2025.1641635 (PMC12575271; doi:10.3389/fcvm.2025.1641635)

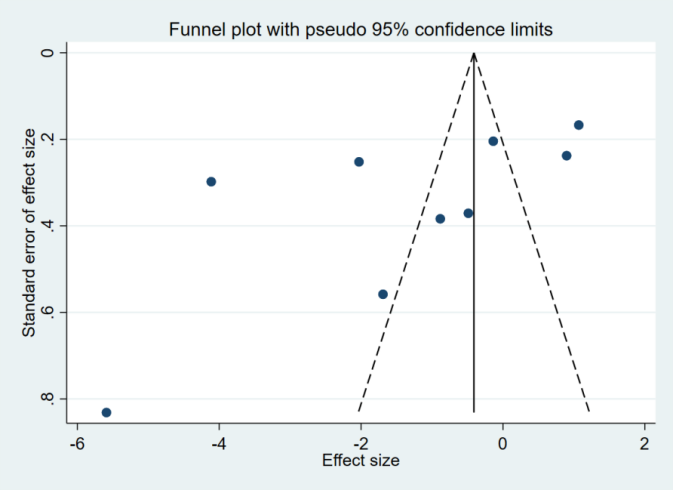

Supplement: Supplementary Figure S1 — Funnel plot of the publication bias of the included studies about biomarkers. [file Image1.tiff]
